# Satisfactory virological response and fibrosis improvement of sofosbuvir-based regimens for Chinese patients with hepatitis C virus genotype 3 infection: results of a real-world cohort study

**DOI:** 10.1186/s12985-018-1066-8

**Published:** 2018-10-01

**Authors:** Ya-Chao Tao, Rong Deng, Meng-Lan Wang, Duo-Duo Lv, Man Yuan, Yong-Hong Wang, En-Qiang Chen, Hong Tang

**Affiliations:** Center of Infectious Diseases, West China Hospital of Sichuan University, No.37 Guo Xue Xiang, Wuhou District, Chengdu, 610041 People’s Republic of China

**Keywords:** Chronic hepatitis C, Hepatitis C virus, Genotype 3, Direct-acting antivirals, Sofosbuvir-based regimens

## Abstract

**Background:**

Chronic hepatitis C virus (HCV) genotype (GT) 3 infection with advanced liver disease has emerged as a challenging to treat by direct-acting antivirals (DAAs), but the efficacy of DAAs in Chinese HCV-GT3 patients is rarely reported. This study aimed to analyze the efficacy of sofosbuvir (SOF)-based regimens in Chinese patients with HCV-GT3 and compensated liver disease.

**Methods:**

This was a registered retrospective study. All patients had completed at least 12 weeks SOF-based regimens therapy (with or without RBV), and were followed up for at least 24 weeks after therapy discontinuation. The primary endpoint was sustained virological response 24 weeks after end of therapy (SVR24).

**Results:**

A total of 102 patients who completed at least 12 weeks therapy were finally included, with 57 in SOF + Daclatasvir (SOF + DCV), 24 in SOF + DCV + ribavirin (RBV) and 21 in SOF/Velpatasvir (SOF/VEL). The total SVR24 rate was achieved in 90.20% (92/102), with 85.96% (49/57) in SOF + DCV, 91.67% (22/24) in SOF + DCV + RBV and 100.00% (21/21) in SOF/VEL. Among 10 relapsed patients (8 in SOF + DCV and 2 in SOF + DCV + RBV), the short course (12 weeks) of therapy and no RBV addition may be the leading cause. In this cohort, the SVR24 rate was not statistically different between patients with and without cirrhosis (81.82% [27/33] vs. 94.20% [65/69], *P* = 0.073). Additionally, both FIB-4 (4.03 vs. 2.08, *P* < 0.001) and APRI (2.15 vs. 0.68, *P* < 0.001) scores were significant improved from baseline to week 24 after completion of therapy, regardless of the presence of cirrhosis.

**Conclusion:**

SOF-based regimens are highly effective in viral clearance and fibrosis remission for Chinese patients with HCV-GT3 infection. If available, SOF/VEL should be first considered.

## Background

Hepatitis C virus (HCV) chronic infection remains a major public health problem [1]. To estimate, there are more than 70 million individuals infected worldwide and 20% of them are infected with genotype (GT) 3 HCV [2]. Patients with HCV-GT3 seem to have a more rapid progression to cirrhosis and hepatocellular carcinoma (HCC) than patients infected with other GTs [3–5]. Though the combination therapy of pegylated-interferon and ribavirin (PR) could bring a higher sustained virological response (SVR) for HCV-GT3 infected patients as compared to HCV-GT1 infected patients, the therapy-related adverse effects are still common and difficult to undergo or unbearable for a consider number of patients [6].

In past years, direct-acting antiviral (DAAs) agents have revolutionized the treatment of CHC, with a significant improvement of efficacy and tolerability [7, 8]. However, in the DAA era, the efficacy of DAAs therapy is lower in HCV-GT3 infected patients than other GT HCV infected patients [8, 9]. Sofosbuvir (SOF), as an NS5B inhibitor, had pan-genotypic activity including effect on HCV-GT3. Thus, SOF-based regimens have been widely used for the therapy of HCV-GT3 infected patients [10–12], and the combination of SOF + RBV was first reported in the therapy of HCV-GT3 chronic infections. After 24 weeks of SOF + RBV therapy, 90% of non-cirrhotic patients could achieve SVR at week 12 after completion of therapy (SVR12), but only 60% of cirrhotic patients could achieve SVR12 [13]. Thus, SOF + RBV combination therapy is not an ideal option for chronic HCV-GT3 infections with cirrhosis.

Recently, more and more DAAs have also approved for CHC therapy, such as daclatasvir (DCV), Ledipasvir (LDV), Velpatasvir (VEL) and Elbasvir/Grazoprevir. And SOF in combination with DCV or VEL has been recommended by EASL guideline for the therapy of HCV-GT3 chronic infections [14]. In fact, the efficacy of SOF plus DCV and VEL-based regimens (SVR12 ≥ 90%) in clinical trials also supports and reinforces their recommendation by guidelines [15–17]. As we all know, chronic HCV infection remain represents a considerable healthcare burden in China [18]. However, until recently, SOF plus DCV and VEL-based regimens are still not approved by China Food and Drug Administration (CFDA). It is worth to mention that, because of the high risk of drug-related adverse reactions, more and more Chinese patients with HCV-GT3 chronic infection refuse to receive PR therapy, and majority of them choose to go abroad for medical treatment or purchase DAAs from overseas pharmacies or hospitals.

Unfortunately, currently data on the efficacy of SOF-based regimens in GT3-infected patients are still limited in China. The aim of this retrospective study is to evaluate the sustained efficacy and safety of SOF-based regimens (DCV or VEL) with or without RBV for HCV-GT3 chronic infections. To our knowledge, this is the first report in a larger real-world cohort of HCV-GT3 chronic infections treated with SOF-based regimens in China.

## Methods

### Study design and patient selection

This is a registered retrospective clinical study in a cohort of HCV GT3-infected patients with compensated liver disease, who received treatment with oral SOF-based regimens in routine clinical practice between January 2016 and May 2017 (Registration number: ChiCTR1800014889). The decision to treat and the choice of DAAs therapy were suggested by the attending physician. And the benefit and potential side effects of DAAs therapy were fully explained to the patient. In this cohort, all DAAs were purchased from overseas pharmacies or hospitals; and all patients had completed at least 12 weeks SOF-based regimens therapy (with or without RBV), and they were followed up for at least 24 weeks after therapy discontinuation.

In this cohort, the therapeutic regimen of DAAs was SOF 400 mg + DCV 60 mg or a fixed-dose combination of SOF400 mg/VEL100mg daily (a single-tablet regimen). The SOF + DCV regimen was administered for either 12 or 24 weeks and included or not a weight-based RBV dose (600–1200 mg/day divided three times daily), depending on each patient’s individual clinical characteristics. This study was conducted in accordance with clinical practice guidelines and was approved by the Ethics Committee of West China Hospital, Sichuan University.

Eligible patients were treatment-naïve patients with chronic HCV GT3 infection, regardless the presence of cirrhosis. In present study, cirrhosis is routinely diagnosed by ultrasound, liver stiffness measurement (FiborScan), and presence of known clinical signs of splenomegaly, hypersplenism and portal hypertension; and all cirrhotic patients were classified as Child-Pugh-Turcotte(CTP) class A at the beginning of therapy. All eligible patients who fulfilled one of the following criteria were excluded: co-infection with other GT HCV; co-infection with hepatitis B virus or human immunodeficiency virus; coexisting serious medical condition or advanced liver disease (including decompensated cirrhosis, liver failure, and hepatic carcinoma); evidence of impairment of renal function.

### Data collection and laboratory examination

The start date of taking DAAs was regarded as the baseline time-point for this study. The general demographic (such as age, gender and route of HCV infection) and clinical laboratory data (such as routine blood test, biochemical parameters, HCV RNA, AFP and liver ultrasound) of patients were regularly collected at baseline, week 4 after therapy, end of therapy, and week 24 after end of therapy.

Serum HCV RNA was determined by RT-PCR of plasma using Cobas AmpliPrep /COBAS TaqMan HCV Test (Roche Diagnostics, Branchburg, NJ), which quantifies HCV RNA with a limit of detection of 15 IU/mL. HCV RNA quantification was performed at baseline, week 4 after therapy, end of therapy, and week 24 after completion of therapy. HCV-genotype was determined in RT-PCR with genotype specific primers from the 5′ non-coding region of the virus. Routine blood test was performed by Automatic Blood Cell Analyzer, and routine biochemical tests were performed by standard procedures (Olympus AU5400, Olympus Corporation, Tokyo, Japan).

### Outcomes and definition

The primary endpoint was sustained virological response (SVR), which was defined as serum HCV RNA undetectable at 24 weeks after the end of therapy in each therapy regimen. The secondary endpoints were the noninvasive scores of liver fibrosis (FIB-4[Fibrosis-4] and APRI [AST to Platelet Ratio Index]), and therapy-related serious adverse event. The scores of FIB-4 and APRI were calculated according to the following formula: FIB-4 = (Age × AST)/(PLT × (square root of ALT)), and APRI = AST/PLT.

Early virological response (EVR) was defined as serum HCV RNA undetectable at week 4 after starting therapy. Virological relapse was established when HCV RNA was detected during follow-up in a patient who had undetectable HCV RNA at the end of therapy. Serious adverse event mainly included hospital admission, hepatic decompensation, HCC occurrence, and death.

### Statistical analysis

Data were presented as mean and 95% confidence interval for normally distributed quantitative variables and as the median and interquartile range for variables with a non-normal distribution. Categorical variables were presented as count and percentage. In present study, serum HCV RNA levels were presented as log transformation. Continuous variables with normal or skewed distribution were analyzed using Student’s or Mann-Whitney test. Paired t-test was used to compare continuous variables before and after therapy. Continuous variables of more than two groups were analyzed using one-way analysis of variance (ANOVA). Categorical variables between groups were analyzed using χ2 test, and Fisher’s exact test when appropriate. The statistical analysis was carried out using the SPSS software package version 16.0 (SPSS Inc., Chicago, IL, USA). A *P*-value of less than 0.05 (two-tailed) was considered to indicate a significant difference.

## Results

### Characteristics of study population

In present study, a total of 113 HCV-GT3 patients were initial screened, 11 patients were excluded and 102 patients were finally included for analysis. Among those 102 HCV-GT3 patients, 57 (55.88%) patients received SOF + DCV therapy, 24(23.53%) patients received SOF + DCV + RBV therapy and 21 (20.59%) patients received SOF/VEL therapy. In this cohort, 33 (32.35%) patients were diagnosed of compensated cirrhosis (CTP class A), with 5(15.15%) patients in SOF + DCV therapy, 23(69.70%) in SOF + DCV + RBV therapy and 5(15.15%) in SOF/VEL therapy. The detailed demographic and clinical characteristics were shown in Table 1.

**Table 1:** Characteristics of patients with GT3-HCV infection who received a sofosbuvir containing treatment regime

	Total	SOF + DCV	SOF + DCV + RBV	SOF+ VEL
Sample size	102	57	24	21
Age	39.99 (38.22–41.76)	39.16 (36.83–41.48)	44.25 (40.8–47.70)	37.38 (33.10–41.67)
Gender (M/F)	62/40	28/29	20/4	13/8
Route of Infection (Travenous drug abuse/Blood products/Others)	58/18/26	34/7/16	10/6/8	14/5/2
Cirrhosis(yes/no)	33/69	4/53	24/0	5/16
Baseline HCV RNA (log10 IU/ml)	6.23 (6.04–6.43)	6.26 (5.99–6.54)	6.33 (5.94–6.73)	6.04 (5.60–6.48)
Baseline ALT (IU/mL)	95.70 (78.88–112.51)	102.67 (78.10–127.24)	112.68 (78.89–146.47)	57.37 (30.01–84.72)
Baseline AST (IU/mL)	81.96 (72.12–91.80)	91.33 (77.48–105.19)	89.86 (71.20–108.52)	47.50 (31.63–63.36)
Baseline TBil (μmol/mL)	19.95 (18.57–21.32)	18.91 (17.22–20.59)	22.01 (18.07–25.94)	20.42 (18.07–22.77)
Baseline PLT (10^9^/L)	142.94 (129.36–156.52)	157.65 (139.05–176.25)	104.96 (79.71–130.21)	146.43 (117.94–174.91)
Baseline FIB-4	4.03 (2.99–5.07)	3.75 (2.27–5.23)	5.70 (3.81–7.58)	2.86 (0.52–5.19)
Baseline APRI	2.15 (1.66–2.64)	2.07 (1.40–2.74)	3.11 (2.02–4.20)	1.26 (0.36–2.16)

### Sustained virological response

**Fig. 1:**
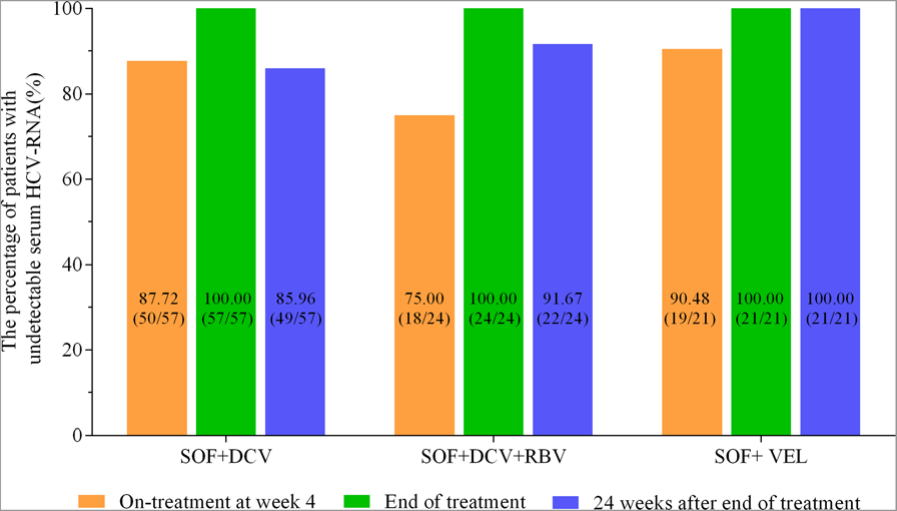
The virological responses at different time-points for CHC patients receiving SOF-based regimens

In the overall cohort, 87 (85.29%) patients achieved EVR, and all 102 (100%) patients had achieved virological response at the end of therapy. At week 24 after completion of therapy, the overall percentage of patients with SVR24 was 90.20% (92/102), which included 85.96% (49/57) in SOF + DCV therapy, 91.67% (22/24) in SOF + DCV + RBV therapy and 100.00% (21/21) in SOF/VEL therapy. Though the absolute percentage of patients with EVR (90.48%[19/21]) and SVR24 (100%[21/21]) in SOF/VEL were both higher than that in SOF + DCV (87.72%[50/57] for EVR and 85.96%[49/57] for SVR24) and SOF + DCV + RBV (75.00%[18/24] for EVR and 91.67% [22/24] for SVR24), the difference was not statistically significant among these SOF-based regimens (*P* = 0.264 for EVR and *P* = 0.226 for SVR24). The detailed virological response during and after therapy were shown in Fig. 1.

**Fig. 2:**
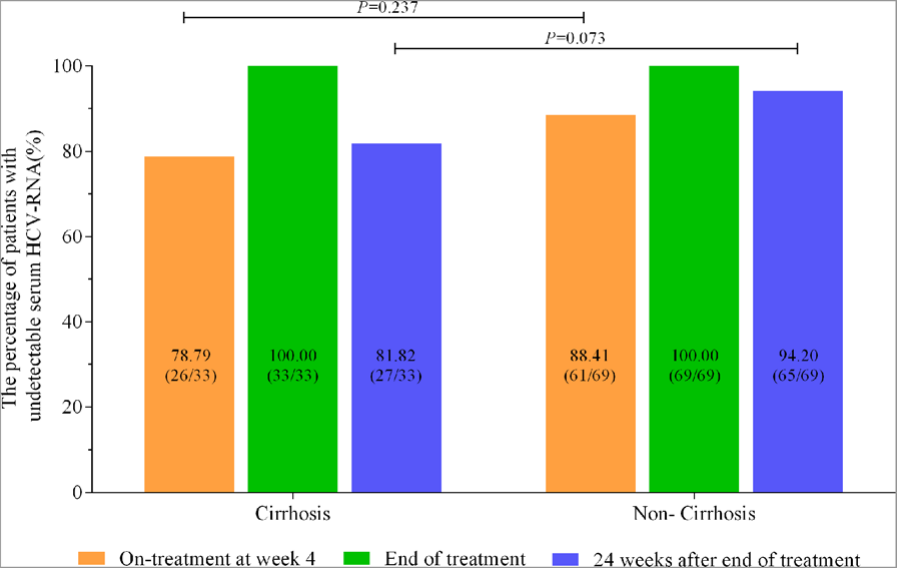
The virological responses at different time-points for CHC patients with and without cirrhosis

In cirrhotic patients, the percentage of patients with EVR and SVR24 was 78.79% (26/33) and 81.82% (27/33), respectively. In non-cirrhotic patients, the percentage of patients with EVR and SVR24 was 88.41% (61/69) and 94.20% (65/69). And the difference in either EVR (*P* = 0.237) or SVR24 (*P* = 0.073) was not statistically significant between cirrhotic and non-cirrhotic patients (Fig. 2).

**Fig. 3:**
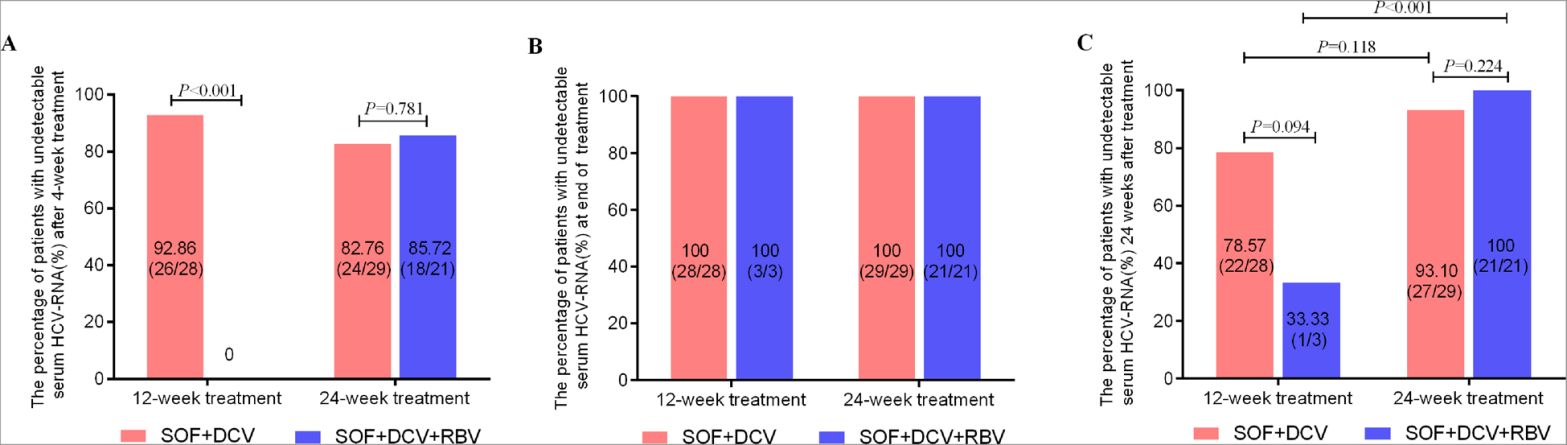
The virological responses in patients receiving 12-week or 24-week SOF + DCV ± RBV therapy

Among the group of SOF + DCV, 28 cases were treated for 12 weeks and 29 cases received therapy for 24 weeks. Whereas, in group of SOF + DCV + RBV, 21 cirrhotic patients underwent 24-week treatment, and 3 cases received 12-week therapy. The virological response was further compared between patients receiving SOF + DCV and SOF + DCV + RBV therapy for 12 weeks and 24 weeks, respectively. As shown in Fig. 3, none achieved EVR with SOF + DCV + RBV after 4-week treatment, and 26 (92.86%) patients achieved EVR after 12-week SOF + DCV treatment, and the difference (*P* < 0.001) between them may relevant to the number and proportion of cirrhotic patients in their respective groups. The percentage of patients with ERV receiving 24-week therapy in two groups was close and showed no significant difference (82.76% [24/29] for SOF + DCV and 85.72% [18/21] for SOF + DCV + RBV, *P* = 0.781). Although the percentage of patients with SVR in SOF + DCV group was higher than that in SOF + DCV + RBV after 12-week therapy, no significant difference was observed (78.57% for SOF + DCV and 33.33% for SOF + DCV + RBV, *P* = 0.094). The difference in SVR24 was not statistically significant between SOF + DCV and SOF + DCV + RBV patients following 24-week therapy (*P* = 0.224). But the percentage of patients with SVR24 was significantly different in patients treated with 12-week and 24-week SOF + DCV + RBV therapy (*P* < 0.001).

In this cohort, 10 patients experienced viral relapse, including 8 patients in SOF + DCV and 2 patients in SOF + DCV + RBV. In the SOF + DCV group, 6 patients were treated for 12 weeks; and the other 2 patients had cirrhosis and were treated for 24 weeks. In the SOF + DCV + RBV group, both two patients had cirrhosis and were treated for 12 weeks. The detailed information of these 10 patients was also shown in Table 2.

**Table 2:** Characteristics of patients with GT3-HCV infection who experienced viral relapse after stopping sofosbuvir containing treatment regime

No.	Age	Gender	Route of Infection	HCV RNA	ALT	AST	TBil	PLT	Cirrhosis	FIB-4	APRI	Treatment	
												Strategy	Actual Duration
1	48	M	Unknown	4.34	73	87	18.4	132	no	3.70	1.65	SOF + DCV	12
2	50	M	Travenous drug abuse	6.30	46	70	16.2	117	no	4.41	1.50	SOF + DCV	12
3	25	F	Travenous drug abuse	6.54	52	84	17.7	152	no	1.92	1.38	SOF + DCV	12
4	73	M	Blood products	4.16	155	217	29.5	32	yes	39.76	16.95	SOF + DCV	24
5	35	M	Travenous drug abuse	6.85	24.6	47.7	28.2	34	yes	9.90	3.51	SOF + DCV + RB	12
6	37	M	Unknown	7.35	72.1	115.8	29	182	yes	2.77	1.59	SOF + DCV + RB	12
7	36	M	Travenous drug abuse	6.98	331	221	29.1	82	yes	5.33	6.74	SOF + DCV	24
8	41	M	Travenous drug abuse	6.53	124	144	17.3	82	yes	6.47	4.399	SOF + DCV	12
9	43	F	Travenous drug abuse	6.00	100	100	13.2	34	yes	12.65	7.35	SOF + DCV	12
10	48	F	Blood products	7.06	81	122.8	12.6	59	no	11.10	5.20	SOF + DCV	12

### Noninvasive evaluation of liver fibrosis

**Fig. 4:**
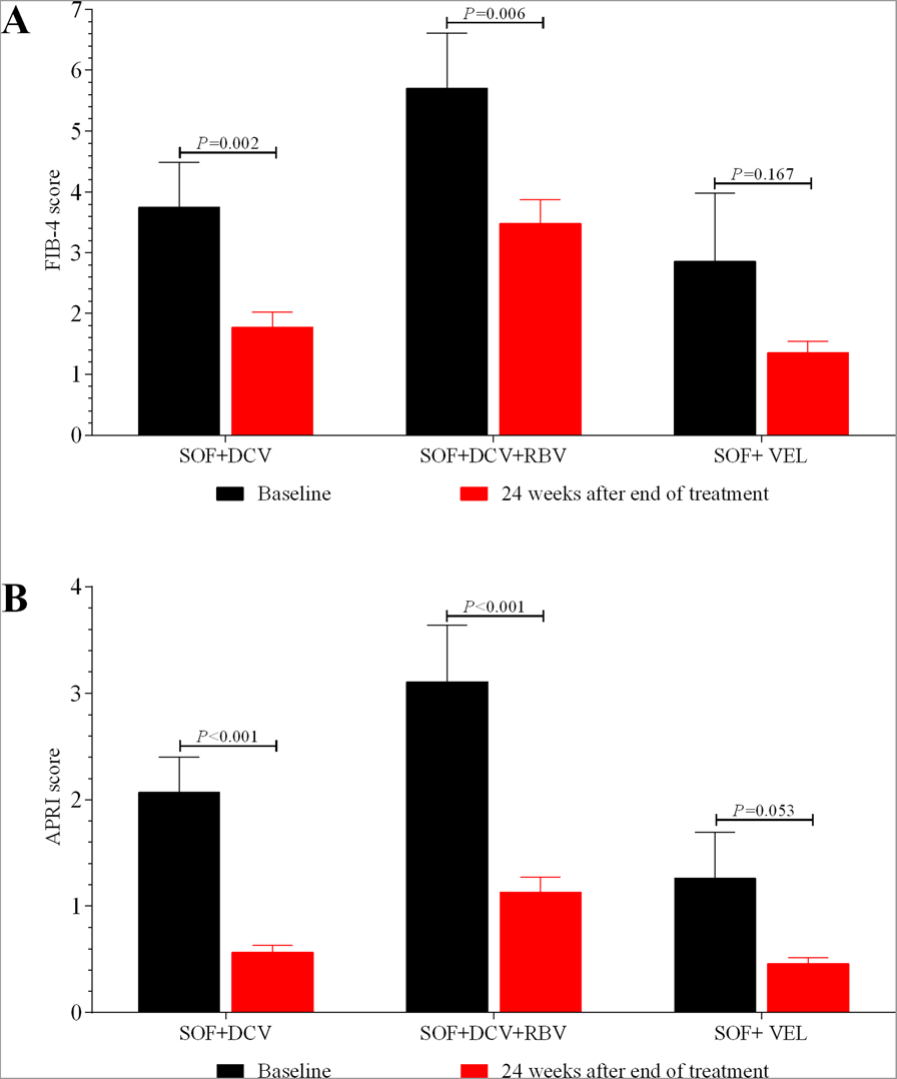
The improvement of FIB-4 (**a**) and APRI (**b**) among patients with receiving SOF-based regimens

As shown in Fig. 4, the mean scores of FIB-4 (4.03 vs. 2.08, *P* < 0.001) and APRI (2.15 vs. 0.68, *P* < 0.001) were both significantly lower at week 24 after completion of therapy as compared to that at baseline. Except for patients with SOF/VEL therapy (from 2.86 to 1.35, *P* = 0.167), the mean scores of FIB-4 in patients with SOF + DCV (from 3.75 to 1.77, *P* = 0.002) and SOF + DCV + RBV (from 5.70 to 3.48, *P* = 0.006) therapies were both significantly reduced at week 24 after completion of therapy as compared to that at baseline. And similar findings of APRI score were also found in SOF + DCV (from 2.07 to 0.57, *P* < 0.001), SOF + DCV + RBV(from 3.11 to 1.13, *P* < 0.001) and SOF/VEL (from 1.26 to 0.46, *P* = 0.053) therapies.

**Fig. 5:**
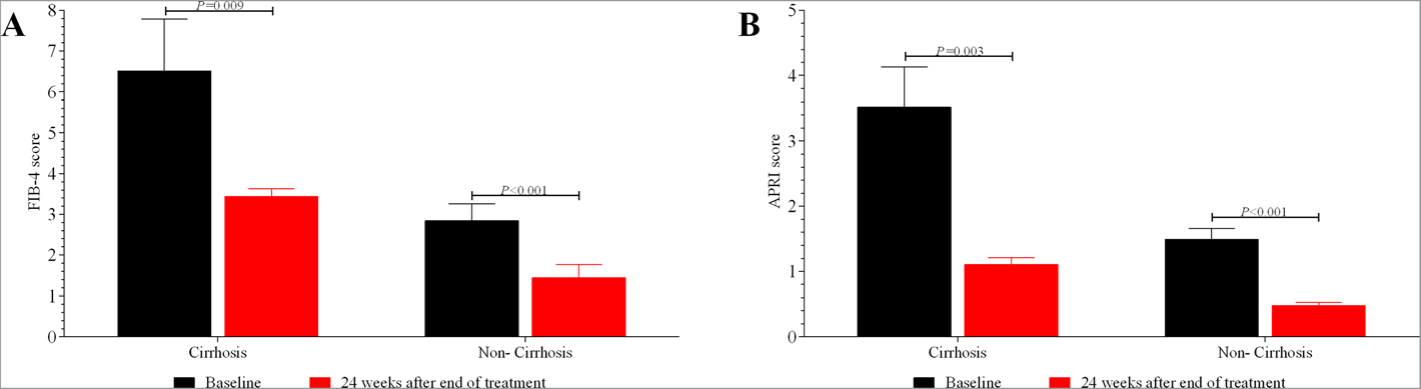
The improvement of FIB-4 (**a**) and APRI (**b**) among patients with and without cirrhosis

As shown in Fig. 5, the mean scores of FIB-4 were significantly reduced from baseline to week 24 after completion of therapy in both cirrhotic patients (from 6.51 to 3.44, *P* = 0.009) and non-cirrhotic patients (from 2.84 to 1.44, *P* < 0.001). Similar findings of APRI were also found in both cirrhotic patients (from 3.52 to 1.10, *P* = 0.003) and non-cirrhotic patients (from 1.49 to 0.48, *P* < 0.001).

**Fig. 6:**
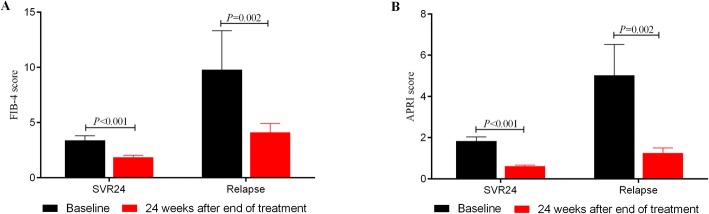
The improvement of FIB-4 (**a**) and APRI (**b**) among patients with SVR24 and virus relapse

We further compared the mean scores of FIB-4 and APRI among patients with SVR24 and recurrence, and found that the mean scores of FIB-4 decreased significantly from baseline to week 24 after completion of treatment in both groups of virological responders and relapsed patients (from 3.399 to 1.863, *P* < 0.001 for patients with SVR12; from 9.801 to 4.123, *P* = 0.002 for relapsed patients, Fig. 6a). The similar performance was also observed in the change of APRI (from 1.834 to 0.619, *P* < 0.001 for patients with SVR12; from 5.026 to 1.248, *P* = 0.002 for relapsed patients, Fig. 6b). It seemed that after DAA antiviral therapy for at least 12 weeks, the fibrosis status of all patients achieved improvement, regardless of their HBV-DNA levels, and the rebound of HCV did not affect the degree of liver fibrosis during our follow-up.

### Serious adverse event

Treatment was well tolerated in all patients, and no serious adverse events occurred during therapy and during follow-up period, and the values of estimated glomerular filtration rate (eGFR) fluctuated within normal range. RBV-related hemolysis was reported in 11(45.83%) patients during SOF + DCV + RBV therapy, and 5 (20.83%) patients had hemoglobin levels less than 100 g/L.

## Discussion

Patients with HCV-GT3 infection are challenge to treat, particularly those who are accompanied with liver cirrhosis [19]. Up to now, the available DAAs for HCV-GT3 infections were still limited. However, the regimens of SOF + DCV ± RBV and SOF/VEL are now recommended by EASL guideline of CHC [14]. The advantage of the present study is that it is one of the few that have evaluated the efficacy of safety of SOF-based regimens in GT3-infected patients in a real-world cohort from China. The main finding of present study was that SOF-based regimens was highly effective in patients with HCV-GT3 infection, and excellent SVR rate was especially found in patients receiving SOF/VEL therapy for 12 weeks. In addition, present study also found that hepatic fibrosis may be alleviated to some extent after HCV eradication.

Because SOF is a nucleotide analogue HCV NS5B polymerase inhibitor with similar in vitro activity against all HCV genotypes, several SOF-based regimens have been reported in the therapy of HCV-GT3 patients, including SOF + RBV, SOF + DCV ± RBV, SOF/LDV ± RBV and SOF/VEL [8, 13, 14, 16, 17, 20]. Additionally, DCV also has shown great potency in vitro against HCV GT3. Thus, the combination of SOF + DCV has been widely concerned for the therapy of HCV-GT3 patients. In past years, the efficacy of SOF + DCV ± RBV regimen for HCV-GT3 patients has been evaluated in two important phase III studies (ALLY-3 and ALLY3+) [16, 17]. In the ALLY-3 study, a total of 101 treatment-naïve patients received SOF + DCV therapy for 12 weeks (without RBV addition), and yielded a 90% overall SVR12 rate. However, the rate of SVR12 decreased from 96% in non-cirrhotic patients to 63% in cirrhotic patients [16]. This data indicated the important effect of cirrhosis on the SVR of SOF + DCV regimen. In present study, we also found that the absolute SVR24 rates with DCV + SOF were higher in non-cirrhotic patients (94.20%) than in cirrhotic patients (81.82%), which were generally consistent with data from ALLY-3 study.

In ALLY 3+ study, patients with advanced fibrosis or cirrhosis further received SOF + DCV + RBV therapy for 12~ 16 weeks, and the SVR12 rate increased to 88% ~ 92% [17]. And the finding indicated that the combination of DCV + SOF, with the addition of RBV, could provide improved response rates for cirrhotic patients. In present study, there were 33 naïve cirrhotic patients, with 72.73% (24/33) in SOF + DCV + RBV, 12.12 (4/33) in SOF + DCV and 15.15% (5/33) in SOF/VEL; the total SVR24 rate was only 81.82% (27/33). However, the SVR24 rate of cirrhotic patients was high to 91.67% (22/24) in SOF + DCV + RBV therapy, including 100% (21/21) receiving 24-week treatment and 33.33% (1/3) receiving 12-week treatment. 2 cirrhotic patients treated with 12-week SOF + DCV + RBV did not achieve SVR24 and experienced viral relapse, demonstrating the importance and necessity of adequate time for cirrhotic patients with SOF + DCV + RBV therapy. Similarly, in all enrolled cirrhotic and non-cirrhotic patients, the SVR24 rate of patients receiving 24-week therapy was a litter higher than that in patients with12-week therapy (84.31% [43 /51] vs. 96.08%[49/51], *P* = 0.047, data not shown). And among 4 cirrhotic patients in SOF + DCV, 3 cirrhotic patients experienced viral relapse during follow-up. So our findings further support and reinforce the recommendation that the addition of RBV could improve the SVR of DCV + SOF therapy for cirrhotic patients with HCV-GT3 infection. Additionally, longer treatment duration of DCV + SOF (for example, 24 weeks) may also help to prevent or decrease the risk of viral relapse in HCV-GT3 infection, especially in those with cirrhosis.

VEL is a new NS5A protein inhibitor with pan­genotypic activity against HCV in vitro, and the fixed-dose combination of SOF/VEL for 12 weeks provide high SVR12 rates in both non-cirrhotic and compensated cirrhotic HCV-GT3 patients in Europe and the United States [11]. In the phaseIIopen-label study by Everson GT et al., 12 week of SOF/VEL has resulted in 93% SVR rates in naïve non­cirrhotic patients [21]. In ASTRAL-3 study reported by Foster GR et al., HCV-GT3 patients who received SOF/VEL therapy, the rate of SVR12 was 95%; and as compared to 97% SVR among non-cirrhotic patients, the rate of SVR was still high to 91% among compensated cirrhotic patients [11]. In this real-world study, 21 SOF/VEL-treated patients, including 5 compensated cirrhotic patients, all achieved SVR12 after a period of 12 weeks of therapy. Additionally, one recent retrospective study forcing on patients with compensated cirrhosis or advanced fibrosis, also reported that the SVR was 95% (145/153) in HCV-GT3 patients with 12 weeks of SOF/VEL therapy [22]. So, SOF/VEL regimen may be a good choice for HCV-GT3 patients, regardless of the presence of cirrhosis.

It is reported that HCV eradication is associated with lower rates of liver fibrosis progression, and early successful therapy could prevent long-term liver complications of HCV infection [23]. Prof. Wei L et al. also reported that the mean risk scores for cirrhosis and HCC were reduced between baseline and follow up in treated patients but not in untreated patients, with the largest reductions seem among treated patients who achieved SVR24 [18]. In present study, we first reported that the value of FIB-4 and APRI improved significantly, which somehow indicated a possibility that hepatic fibrosis may be alleviated after effective control of HCV RNA. However, the evidences of liver biopsy or non-invasive assessment of transient elastography (FibroScan) are badly needed to further confirm the liver histology improvement of DAAs therapy.

As with all real-world studies, present study also has some limitations. For example, the diagnosis of cirrhosis was based on clinical criteria, rather than on the current “gold standard” of histology. This study was a retrospective study, and patients who had poor treatment responses were easy to loss, and this would bias the results of virological responses at the end of therapy. Additionally, the sample size in our study was relatively small and it was also not comparable among each group.

## Conclusion

HCV GT3 patients should no longer be considered as a difficult- to- treat subgroup at present. The SOF-based regimens were highly effective in viral clearance and fibrosis remission for patients with HCV-GT3 infection. If available, 12 weeks of SOF/VEL should be first considered, regardless of the presence of cirrhosis.

## Abbreviations

APRI: AST to Platelet Ratio Index
CHC: Chronic hepatitis C
DAAs: Direct-acting antivirals
DCV: Daclatasvir
eGFR: Estimated glomerular filtration rate
FIB-4: Fibrosis-4
GT: Genotype
HCC: Hepatocellular carcinoma
HCV: Hepatitis C virus
RBV: Ribavirin
SOF: Sofosbuvir
SVR: Sustained virological response
VEL: Velpatasvir
